# Food insecurity and nutritional status of preconception women in a rural population of North Karnataka, India

**DOI:** 10.1186/s12978-018-0535-2

**Published:** 2018-06-22

**Authors:** Shivanand C. Mastiholi, Manjunath S. Somannavar, Sunil S. Vernekar, S. Yogesh Kumar, Sangappa M. Dhaded, Veena R. Herekar, Rebecca L. Lander, Michael K. Hambidge, Nancy F. Krebs, Shivaprasad S. Goudar

**Affiliations:** 1 Women’s and Children’s Health Research Unit, KLE Academy of Higher Education and Research’s Jawaharlal Nehru Medical College, Belagavi, Karnataka India; 2 Department of Pediatrics, Section of Nutrition, University of Colorado School of Medicine, Aurora, CO USA

**Keywords:** Preconception women, Nutritional status, Socio-economic status and food insecurity

## Abstract

**Background:**

As per the World Health Organization, the nutritional status of women of reproductive age is important, as effects of undernutrition are propagated to future generations. More than one-third of Indian women in the reproductive age group are in a state of chronic nutritional deficiency during the preconception period leading to poor health and likely resulting in low birth weight babies. This study was aimed to assess the food insecurity and nutritional status of preconception women in a rural population of north Karnataka.

**Methods:**

A total of 770 preconception women were enrolled across a district in Karnataka from selected primary health centre areas by a cluster sampling method. Data on socioeconomic status, food insecurity and obstetric history were collected by trained research assistants, interviewing women at home. In half of the participants, a 1 day 24 –hour dietary recalls were conducted by dietary assistants to assess the dietary intakes. Anthropometric measurements and haemoglobin estimation were carried out at the health centres.

**Results:**

In the present study, a majority of the participants (64.8%) belonged to the lower socio-economic classes and the prevalence of food insecurity was 27.4%. A majority of the participants had mild (15.5%) to moderate (78.6%) anaemia. About one-third of the participants (36.6%) were underweight. Significant associations were found between socio-economic status and anaemia (*p* = 0.0006) and between food insecurity and anaemia (*p* = 0.0001).

**Conclusion:**

The nutritional status of preconception women was poor and anemia was more prevalent in low-socioeconomic and food insecure population.

## Background

Malnutrition, especially undernutrition is prevalent in developing countries and the adverse effects of poor nutrition on pregnancy outcomes have been well documented. [1] Reproductive-aged women are at risk of iron deficiency because of blood loss from menstruation, poor diet, and frequent pregnancies [2]. Resource poor settings like in India affect the health and nutritional status of women of reproductive age exacerbated by prevailing cultural and traditional practices. [3, 4] Women are at high risk of inadequate micronutrient intakes as their diets are of low-quality, lack diversity and are dominated by staple foods. [5] In India, like other low resource settings, women are vulnerable to undernutrition for social and biological reasons throughout their life-cycle. [6, 7]

Maternal nutritional status is important for the health and quality of life of women and for the health of their newborns. India is home to more than 217 million undernourished people as per The State of Food Insecurity in the World (2012) estimates [8]. Previous studies in Indian urban settings have found the prevalence of food insecurity ranging from 51 to 77% [9–12]. However, data on food insecurity in the rural areas is lacking which constitutes about 70% of Indian population. [13] This study was aimed to assess the food insecurity, socio demographic factors, nutritional status, macro and micronutrient adequacy of diets in the preconception period among rural women of North Karnataka.

## Methods

This was a cross sectional study conducted from January to December 2014. A total of 770 preconception women were enrolled belonging to 18 villages from five counties across the Belagavi district in Karnataka state. Women were identified through a household survey and selected by a cluster sampling method with the help of Nurse Midwives and Accredited Social Health Activists (ASHAs). Non-pregnant and non-lactating women with parity 0–3 were included. Those with haemoglobin less than 8 g/dL and/or who were using permanent and temporary methods of birth control were excluded. The study was approved by J N Medical College, Belagavi Institutional Ethics Committee on Human Subjects Research. Informed written consent was obtained from each study participant.

Socio-demographic, obstetric and food insecurity data were collected by trained Home Visit Research Assistants (HVRA) by interviewing women in their households. Information regarding per capita income (in Rupees/month) was collected and socio-economic status (SES) was classified using the Modified Prasad classification for the study period (2014). SES was classified as upper, upper middle, middle, lower middle and lower Class based on per capita monthly income of Rupees 5357 and above, 2652–5356, 1570–2651, 812–1569 and 811 or less respectively [14] Information regarding parity, age of marriage and history of consanguinity (matrimony between closely related individuals) were collected as part of obstetric history. Food insecurity status was assessed based on a nine item questionnaire and categorized into four types: food secure, mildly food insecure, moderately food insecure and severe food insecure using Household Food Insecurity Access Scale (HFIAS) for measurement of food access [15].

Hemoglobin (Sahli’s Method) [16] was estimated by trained technicians using capillary blood in the health centres and anaemia was graded as per WHO criteria [17]. The anthropometric measurements were carried out in primary health centres. Maternal height, weight, mid-upper arm circumference (MUAC), waist, and hip circumference measurements were obtained by a specially trained assessment team utilizing standardized calibrated study equipment. Subjects were lightly clothed with no footwear. Height was recorded to the nearest 0.1 cm and weight to the nearest 0.1 kg by utilizing stadiometer and electronic weighing scales respectively. MUAC and, Waist and Hip Circumferences were recorded to the nearest 0.1 cm by Circumference insertion measuring tape and retractable tapes respectively. BMI (kg/m^2^) was calculated from recorded height and weight. The WHO recommended appropriate Body Mass Index (BMI) for Asian populations and their cut-off values were used for classification. Women were classified as underweight, Normal, Overweight and Obese based on BMI of less than 18.5, 18.5 to 22.9, 23 to 24.9 and more than or equal to 25 respectively. [18–20]

One day 24-h dietary recall was conducted by the trained dietary assistants in half of the study participants to estimate the intake of macro and micronutrients. Additionally, in 30% of the study participants, repeat dietary recall was performed to validate the nutritional intakes (Data not shown). The method employed for the 24-h dietary recall is published elsewhere [21].

Socio-demographic and food insecurity variables were presented as descriptive statistics. Nutrient intakes and anthropometric parameters were summarized as means and standard deviations. Chi-square tests were used to define the association between various socio-demographic variables and food insecurity, Anemia and poor nutrient intake (proportion of women with less than 50% intake of recommended daily allowance) with a significance level of 0.05. Data was entered in excel to prepare a master chart. SPSS version 21.0 software was used for analysis of the data.

## Results

**Fig. 1:**
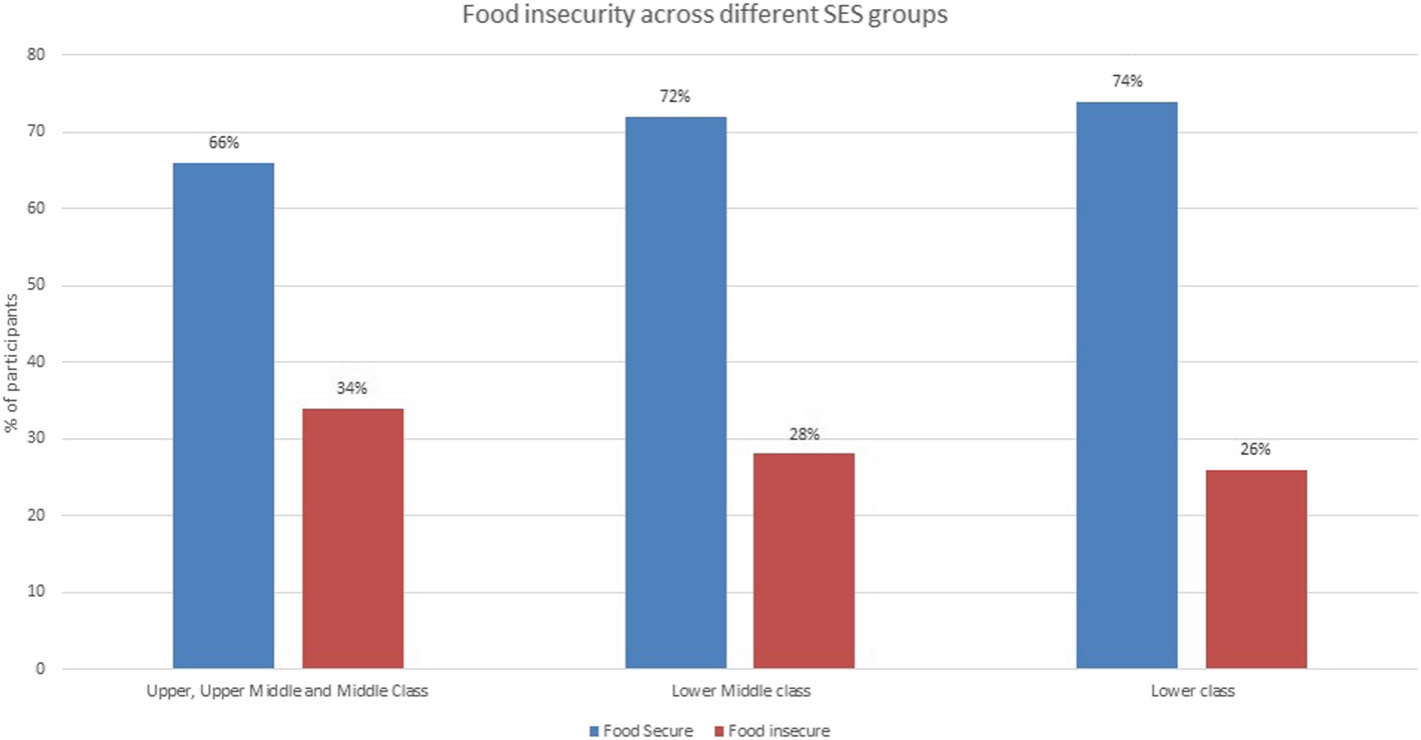
Prevalence of Food insecurity among different Socioeconomic classes

A total of 770 participants were included in the study. The mean age of the participants was 22.5 (SD ± 3.19) years and the majority (93.6%) were less than 30 years. The majority of participants (84.3%) were Hindus whereas Muslims constituted 15.5%. Almost two-third of the participants (65%) had studied at a secondary or higher level. The majority of the women (89%) were classified as low or lower middle socio-economic class. About 38% of the participants had a history of consanguineous marriage and slightly more than one third were married before the age of 18 years (37.8%). Forty-four percent of the preconception women had one child and 33.5% were nulliparas. Almost 94% of the women were anaemic with 78.6% and 15.5% classified as moderate and mild anemia respectively. Nearly a quarter of households were mild to moderate food insecure (27.4%) with severe food insecurity among 4.6% (Table 1) Food insecurity was prevalent across all the SES categories. It was found to be 33.7% in upper, upper-middle and middle classes, 28.1% in lower middle classes and 26.1% in lower class families but the differences were not statistically significant (*p* = 0.4334). (Fig. 1).

**Table 1:** Socio-demographic parameters, Food Security and Anaemic status of preconception women

Variables		N	Percentage
Age:	15–19 years	126	16.4
	20–24 years	436	56.6
	25–29 years	174	22.6
	30–34 years	28	3.6
	35–39 years	6	0.8
Religion:	Hindu	649	84.3
	Muslim	119	15.5
	Others	2	0.3
Education:	Illiterate	52	6.8
	Primary	228	29.5
	Secondary	331	43.0
	PUC	123	16.0
	Graduate/Post graduate	36	4.7
Socio economic status:	Upper class	6	0.8
	Upper Middle class	22	2.9
	Middle class	58	7.5
	Lower Middle class	185	24.0
	Lower class	499	64.8
Lacto-Vegetarian Diet:		375	48.7
Age of marriage: < 18		291	37.8
Consanguinity (Yes)		290	37.7
Parity:	Nulliparous	258	33.5
	1 child	338	43.9
	2 child	141	18.3
	3 + child	33	4.3
Hemoglobin: G/dL	Moderate Anemia (8–10.9)	605	78.6%
	Mild Anemia (11–11.9)	119	15.5%
	Normal (≥12)	46	5.9%
Household food insecurity access (HFIA) category	Food secure (HFAI 1)	559	72.6
	Mild food insecure (HFAI 2)	84	10.9
	Moderate food insecure (HFAI 3)	92	11.9
	Severe food insecure (HFAI 4)	35	04.6

One third of the study participants (36.6%) were underweight and about 18% were either overweight (8.7%) or obese (9.1%). The mean MUAC of the participants was 24.1 cm. Nearly 25% of the participants had a MUAC less than 22.0 cm. A total of 180 (23.4%) participants had W/H ratio more than or equal to 0.8 (Table 2).

**Table 2:** Anthropometric parameters

Variables	N (%) (*n* = 770)	Mean ± SD
Height (in cm)		151.42 ± 5.58
Weight (in kg)		46.212 ± 8.4398
BMI: (Kg/m^2^)		20.09 ± 3.39
Underweight (< 18.5)	282 (36.6%)	
Normal (18.5–22.9)	351 (45.6%)	
Overweight (23–24.9)	67 (8.7%)	
Obese ≥25	70 (9.1%)	
Waist circumference (cm)		65.18 ± 8.44
Hip circumference (cm)		85.22 ± 7.14
W/H ratio		0.76 ± 0.06
< 0.8	590 (76.6%)	
≥ 0.8	180 (23.4%)	
MUAC (cm)		24.06 ± 3.06
< 22	189 (24.5%)	
≥ 22	581 (75.5%)	

The mean and median intakes of energy, macro and micronutrients are presented in Table 3. With respect to the energy and macronutrients, the proportion of women consuming less than 50% of the RDA were 15.8% for energy, 39.6% for protein and 18.2% for lipids. The consumption deficit was even greater for micronutrients. The proportion of women taking less than half of the required daily allowance for calcium, iron, zinc, vitamin B12, folate, vitamin C and vitamin A was 32.8%, 77.7, 54.5, 35.4, 43.9, 38.5 and 44.5% respectively.

**Table 3:** Calorie and nutrient intake on selected Macro and micronutrients (*n* = 392)

Nutrient (RDA)	Mean ± SD	Median	Proportion of women with intake < 50% of RDA
Calorie (Kcal) (1900 kcal)	1302.66 ± 358.50	1263.32	15.8
Protein (g) (55 g)	31.24 ± 11.07	29.77	39.6
CHO (g) (280 g)	183.64 ± 49.42	180.67	1.0
Total lipids (Fat)(g) (40 g)	50.47 ± 17.78	48.72	18.2
Fiber (g) (30 g)	15.57 ± 5.25	14.97	49.8
Calcium(mg) (600 mg)	453.68 ± 252.16	389.59	32.8
Iron(mg) (21 mg)	8.20 ± 3.04	7.82	77.7
Zinc(mg) (10 mg)	5.04 ± 1.76	4.79	54.5
Vit B1(Thiamine)(mg) (1 mg)	0.51 ± 0.18	0.50	49.0
Vit B2 (Riboflavin)(mg) (1.1 mg)	0.75 ± 0.29	0.72	24.2
Vit B6 (Pyridoxal phosphate PLP)(mg) (2 mg)	0.97 ± 0.34	0.95	57.6
Dietary folate equiv.(DFE)(μg) (200 μg)	125.16 ± 67.03	112.35	43.9
Vit B12 (Cobalamin)(μg) (1 μg)	0.92 ± 0.80	0.621	35.4
Vit C (Ascorbic acid)(mg) (40 mg)	30.33 ± 22.54	24.69	38.5
Vit A RAE (μg) (400 μg)	266.54 ± 178.26	216.28	44.5

**Fig. 2:**
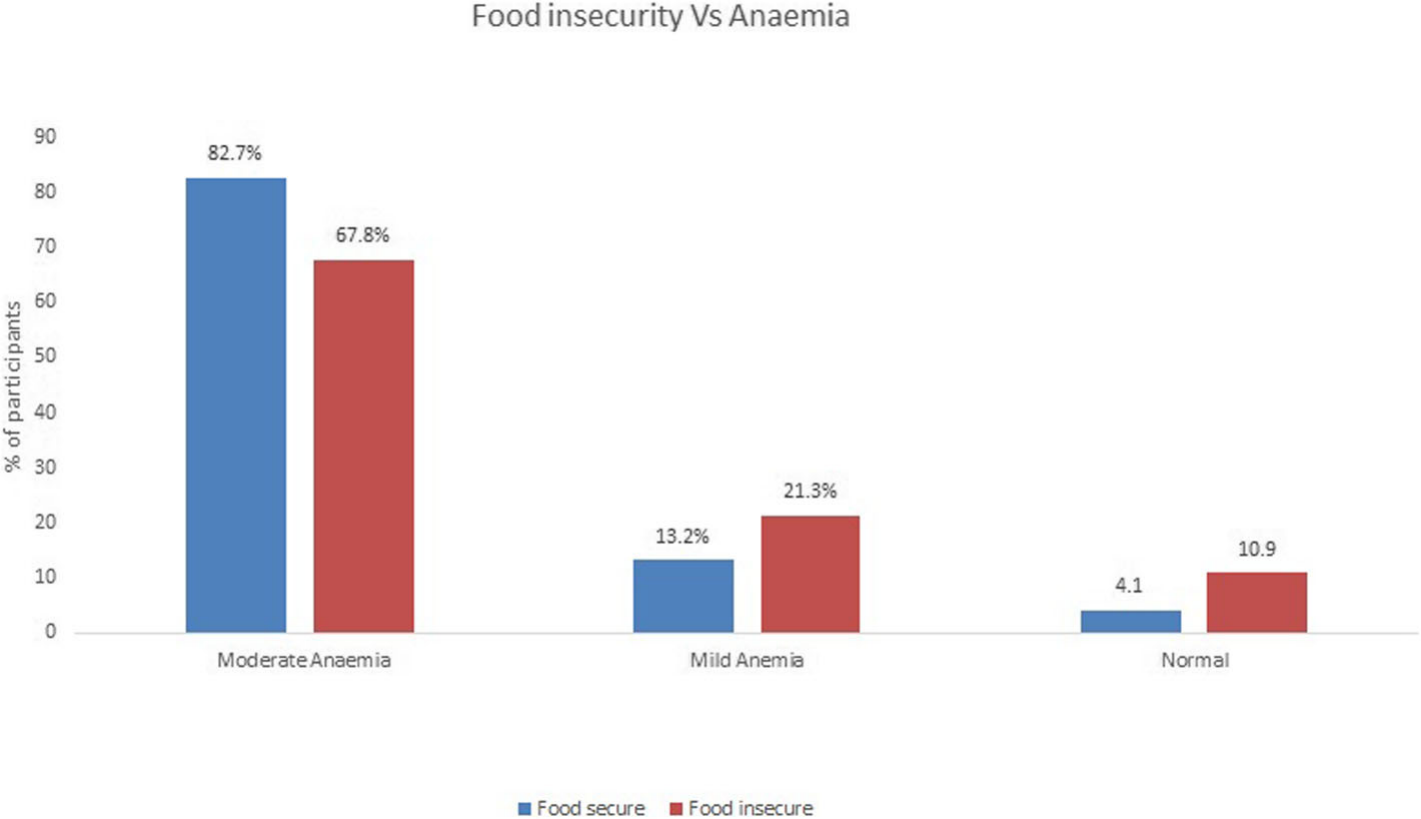
Prevalence of Anemia among food secure and insecure women

The anaemia prevalence was 94.1% and found to be present in women across all the SES categories. About 70% of the women from upper, upper-middle and middle income families had moderate anaemia whereas prevalence was more in lower-middle (73%) and lower (82.2%) income families. Mild anemia was observed in 27.9% of upper, upper-middle and middle compared to 18.9 and 12% in lower-middle and lower income families respectively. The severity of anaemia was greater in lower SES categories and the association was statistically significant (*p* = 0.0006). (Table 4). Anaemia was more common in food secure (95.8%) than in food insecure (89.1%) women. Prevalence of moderate anemia was higher (82.6%) in food secure than in food insecure (67.8%) women. However, mild anemia was more prevalent in food insecure (21.3% vs 13.2%) women and the results were statistically significant. (*p* = 0.00001) (Fig. 2). The prevalence of moderate anaemia was slightly lower in Lacto-vegetarians (75.2%) than non-vegetarians (81.1%), whereas mild anemia was more prevalent in Lacto-vegetarians (18.5% vs 12.7%), which was not statistically significant (p = 0. 067771). (Table 5) The association between dietary intake of micronutrients (iron, vitamin B6, folate and vitamin B12) and anaemia in vegetarians and non-vegetarians is presented in Table 6. There were no statistical differences in intake of these micronutrients and anaemia status in both the vegetarian and non-vegetarian groups.

**Table 4:** Prevalence of anemia among different Socioeconomic classes

Socio economic status (SES)	Anaemia status			Total
	Moderate Anaemia	Mild Anaemia	Normal	
Upper, Upper Middle and Middle Class	60 (69.8%)	24 (27.9%)	2 (2.3%)	86
Lower Middle class	135 (73.0%)	35 (18.9%)	15 (8.1%)	185
Lower class	410 (82.2%)	60 (12%)	29 (5.8%)	499
Total	605	119	46	770
X^2^ = 19.6201 DF = 4 p = 0.0006

**Table 5:** Association between type of diet and anemia

	Moderate Anemia	Mild Anemia	Normal	Total
Lacto-vegetarians	282 (75.2%)	69 (18.5%)	24 (6.3%)	375
Non-vegetarians	323 (81.1%)	50 (12.7%)	22 (6.2%)	395
Total	605	119	46	770

**Table 6:** Association between dietary intake of micronutrients and anemia in vegetarians and Non-vegetarians

		Iron		Vitamin B6		Folate		Vitamin B12	
		Women with intake (n)							
		< 50% of RDA	> 50% of RDA	< 50% of RDA	> 50% of RDA	< 50% of RDA	> 50% of RDA	< 50% of RDA	> 50% of RDA
Vegetarians	Normal	11	05	07	09	07	09	05	11
	Anemic	152	39	109	82	87	104	74	117
		X^2^ = 0.489 *p* = 0.4845		X^2^ = 1.063 *p* = 0.3025		X^2^ = 0.019 *p* = 0.8895		X^2^ = 0.351 *p* = 0.5534	
Non-vegetarians	Normal	14	03	11	06	08	09	05	12
	Anemic	143	25	93	75	67	101	65	103
		X^2^ = 0.092 *p* = 0.7617		X^2^ = 0.548 *p* = 0.4591		X^2^ = 0.330 *p* = 0.5657		X^2^ = 0.565 *p* = 0.4522	

## Discussion

The results of the study indicate that nutritional status of preconception women is suboptimal although food insecurity does not appear to be a major concern in this population. Almost all of the women were anemic, about one-third underweight, majority had inadequate intake of micro and macronutrients and one-fourth were food insecure.

This was a community based study with participants identified through household survey of Married Women of Reproductive Age from a cross section of rural areas of Belagavi District of North Karnataka region of India. Food insecurity as well as socioeconomic and nutritional status were determined using validated assessment methods. Additionally, actual intake of macro and micronutrients was assessed by a 24-h dietary recall by a trained dietician. Though less accurate, Haemoglobin level of the participants was estimated by Sahli’s method since the Primary Health Centres generally use this method and is recommended for community screening of anemia. [22, 23] Further, type of anemia was not evaluated in this study. Additionally, clustering was not accounted for in the analysis.

Studies conducted in central and eastern India have reported the prevalence of mild to moderate anaemia in the range of 42.1 to 60.8% and 39.6 to 48.0% respectively. [24, 25] However, National Family Health Survey-2015-16 (NFHS-4) reported a lower prevalence of anaemia (41.2% for Belagavi district and 46.1% for Karnataka state). [26, 27] The lower prevalence may be due to methodological differences in the estimation of Hemoglobin. We found that anaemia was prevalent among all the women irrespective of their socio-economic strata, however, the severity of anaemia was greater among lower SES women. Anthropometric evaluation showed that most of the women had either a normal BMI or were underweight. A study done in a South Indian state [28] showed that 31.3% of preconception women were undernourished and NFHS-4 reported a comparatively lower prevalence (20.6% for Belagavi District and 24.3% for Karnataka state) [26, 27]. Another study in neighbouring Bangladesh [29] showed that 25.6% of preconception women, especially adolescents were undernourished.

Evaluation of the recommended daily allowance and the actual intake of various macro and micronutrients showed that the diet was rich in carbohydrates and fats. The food consumption pattern in this population showed that the caloric intake was relatively adequate in majority of the women. However, the total protein, fibre and essential micronutrient intake was low in the study participants. Earlier studies have reported poor nutritional status in women of reproductive age. [5, 30–34] In the present study, nearly half the participants were Lacto-vegetarians and the type of diet was not associated with prevalence of anaemia. Additionally, intake of important hematopoietic micronutrients such as Iron, Vitamin B6, Folate and Vitamin B12 was not associated with prevalence of anaemia in both the vegetarian and non-vegetarian population. Hence, it may be inferred that vegetarianism may not be a major contributing factor to cause anaemia.

Food insecurity was not a major concern in the study group which was found only in 27.4% families compared to reported prevalence among urban and tribal communities of India. [9–12] Similar findings were observed in a study conducted in north India where 25% of the households were food insecure. [35] A study conducted in the Western Highlands of Guatemala [36] revealed that, 35.9% of women were food secure, 46.1% had moderate food insecurity and 18% women had severe food insecurity. Regardless of the reported food security status, more than three-fourth of women in both food secure and insecure groups were anaemic. It was observed that food security was not a protective factor to prevent women from being anaemic. This can be attributed to the dietary and cultural norms in the society wherein most of the population is vegetarian and where the diet is protein deficit. Another issue is that the society is male dominated where females eat the left over, less nutritious food. We can safely infer that food security does not ensure food quality. Higher food security among low socio-economic strata families compared to lower middle and upper classes can be attributed to various schemes of government like provision of food grains (Rice, Wheat and Finger Millet), sugar and palm oils free of cost or at a highly subsidized rate for low socioeconomic people in the society. [37] Preconception care can make a useful contribution to reducing maternal and childhood morbidity and mortality and also improve maternal and child health in both high- and low-income countries. [38] Results of this study are important as the nutritional status of the preconception women in this population is poor and might impact the health of both mother and child.

## Conclusion

The nutritional status of preconception women is poor and food security is common in this population. Anaemia in women of reproductive age group can be a major public health hazard if not addressed strongly. The health of a woman in the reproductive age group has direct implications on the health of the new born babies she will bear in the future. Though emphasis is given for nutrition during pregnancy, there is an urgent need to create awareness and implement interventions to improve the nutritional status during the preconception period itself so as to improve the maternal health and in turn child health.

## Abbreviations

ASHA: Accredited social health activist
BMI: Body mass index
HFIAS: Household food insecurity access scale
HVRA: Home visit research assistant
MUAC: Mid upper arm circumference
